# Transient Neonatal Hypothyroidism Followed by Hyperthyroidism Due to Maternal Thyrotropin Receptor Antibodies

**DOI:** 10.1210/jcemcr/luaf040

**Published:** 2025-03-07

**Authors:** Mark Garrelfs, Gerdine A Kamp, A S Paul van Trotsenburg

**Affiliations:** Department of Pediatric Endocrinology, Amsterdam University Medical Centers, University of Amsterdam, 1105 AZ Amsterdam, The Netherlands; Department of Endocrinology and Metabolism, Amsterdam Gastroenterology Endocrinology Metabolism, Amsterdam University Medical Centers, University of Amsterdam, 1105 AZ Amsterdam, The Netherlands; Department of Pediatrics, Tergooi Hospitals, 1261 AN Blaricum, The Netherlands; Department of Pediatric Endocrinology, Amsterdam University Medical Centers, University of Amsterdam, 1105 AZ Amsterdam, The Netherlands; Department of Endocrinology and Metabolism, Amsterdam Gastroenterology Endocrinology Metabolism, Amsterdam University Medical Centers, University of Amsterdam, 1105 AZ Amsterdam, The Netherlands

**Keywords:** TSH receptor antibodies, neonatal hypothyroidism, neonatal hyperthyroidism, maternal Hashimoto disease, maternal Graves’ disease

## Abstract

Maternal thyroid dysfunction can negatively influence fetal and/or neonatal thyroid hormone homeostasis. Autoantibodies associated with autoimmune thyroid disease can cross the placenta. TSH receptor antibodies (TRAbs) can either stimulate or block the TSH receptor, and both types of antibodies can be present in the same person. TRAbs are the most important antibodies in Graves’ disease but are also found in a percentage of women with Hashimoto disease. Properties of the dominant TRAb type (stimulating or blocking) will generally dictate the clinical picture. We describe a rare case of neonatal hypothyroidism followed by hyperthyroidism caused by maternal TRAbs, associated with Hashimoto disease. In contrast to similar cases, the mother was not treated with antithyroid drugs, providing evidence for the gradually changing balance between blocking and stimulating TRAbs after birth and their different effects on neonatal thyroid function. This case highlights the need for regular thyroid function tests in neonates with high TRAb titers until maternal antibodies are cleared.

## Introduction

Maternal thyroid dysfunction can negatively affect fetal and neonatal thyroid function [1]. In the first half of pregnancy, before the fetal hypothalamus-pituitary-thyroid axis becomes functional, the fetus is dependent on maternal thyroid hormone acquired through transplacental transport [1-3]. Maternal hypo- or hyperthyroidism can therefore lead to suboptimal fetal thyroid hormone levels and negatively impact neurocognitive development.

Fetal or neonatal thyroid hormone homeostasis can also be disturbed by antithyroid drugs (ATDs) for treatment of maternal hyperthyroidism, or by TSH receptor antibodies (TRAbs) associated with maternal autoimmune thyroid disease [1, 3].

TRAbs can either stimulate the TSH receptor through TSH-stimulating antibodies (TSAb) or block it through thyrotropin blocking antibodies (TBAb). Most assays do not discriminate between TSAb and TBAb and only report the TRAb concentration (ie, antibodies that block binding of naturally occurring TSH to the TSH receptor without determining its stimulating or blocking properties) [4]. TRAbs are the primary antibody in Graves’ disease [5]. However, TRAbs are also found in 10% to 20% of patients with Hashimoto disease [1, 6, 7]. These antibodies can cross the placenta and are associated with fetal and/or neonatal hyper- or hypothyroidism [1, 3, 7]. As both types of antibodies can be present in the same person and their relative proportions can change over time, the effect on offspring is determined by the balance between stimulating and blocking antibodies [8, 9].

Transient fetal and/or neonatal hyperthyroidism is seen in approximately 1% of pregnancies/offspring of mothers with a medical history of Graves’ disease but has also been sporadically described in the offspring of patients with TRAb-positive Hashimoto disease [1, 10, 11]. The incidence of transient hypothyroidism caused by TBAb, mainly but not exclusively associated with Hashimoto disease, is thought to be very low (between 1:84 700 and 1:310 000) [12-15].

We report on a rare case of transient neonatal hypothyroidism followed by transient hyperthyroidism, caused by maternal TRAbs associated with Hashimoto disease. This case is unique in the fact that the mother did not use any ATDs, proving that the changing balance between TRAbs with different properties was the culprit.

## Case Presentation

The male patient is the first child of nonconsanguineous parents of Dutch origin. One year prior to conception, the mother was diagnosed with primary hypothyroidism caused by Hashimoto disease. At diagnosis she had a serum TSH concentration of 260 mIU/L (260 µU/mL) (reference range: 0.6-8 mIU/L; 0.6-8 µU/mL) and a free T4 (FT4) concentration below the assay's detection limit (cutoff: 5.41 pmol/L; 0.42 ng/dL). She had thyroid peroxidase antibody titers of >2000 kIU/L (>2000 IU/mL) (cutoff: < 60 kIU/L; < 60 IU/mL) and TRAb titers >68 IU/L (>68 mIU/mL) (cutoff: < 3.4 IU/L; < 3.4 mIU/mL), both above the assay's detection limit. It is unclear why TRAbs were measured as this is generally not recommended in pregnant women with primary hypothyroidism [16]. No ultrasound of her thyroid was performed. The mother was started on levothyroxine treatment, and with a dose of 200 μg/day she remained euthyroid throughout pregnancy. She never experienced signs or symptoms of hyperthyroidism and had not used ATDs.

Ultrasonographic follow-up during pregnancy revealed normal fetal growth and heart rate, without signs of fetal goiter. TRAb titers remained above the assay's detection limit in the third trimester. As the risk of fetal/neonatal thyroid dysfunction was considered high because of TRAb titers ≥3.7 times the upper limit of normal, labor was induced at 37 weeks.

## Diagnostic Assessment

The patient was born at a gestational age of 37 2/7 weeks. His birthweight was 2875 grams (p31). Physical examination revealed no congenital abnormalities, goiter, proptosis, or signs of thyroid dysfunction like tachycardia.

Cord blood analyses showed a high serum TSH and low FT4 concentration (Table 1). The TRAb titer in cord blood was extremely high (Table 1). As the mother was not on ATDs, a preliminary diagnosis of primary hypothyroidism caused by TBAbs was made.

**Table 1.** Sequential FT4, TSH, and TRAb measurements and medication used

Day	FT4 reference range: 11-22 pmol/L (0.85-1.70 ng/dL)	TSH reference range: 0.6-8 mIU/L (0.6-8 µIU/mL)	TRAb reference range: < 3.4 IU/L (<3.4 mIU/mL)	Levothyroxine μg/day	Thiamazole mg/day
0	10 pmol/L (0.77 ng/dL)	170 mIU/L (170 µIU/mL)	>68 IU/L (>68 mIU/mL)	—	—
3	—	—	—	31.25	—
5	13 pmol/L (1.00 ng/dL)	210 mIU/L (210 µIU/mL)	—	31.25	—
8	20 pmol/L (1.54 ng/dL)	77 mIU/L (77 µIU/mL)	—	31.25	—
14	31 pmol/L (2.39 ng/dL)	2 mIU/L (2 µIU/mL)	>68 IU/L (>68 mIU/mL)	25	—
21	25 pmol/L (1.93 ng/dL)	0.29 mIU/L (0.29 µIU/mL)	—	25	—
28	24 pmol/L (1.85 ng/dL)	0.087 mIU/L (0.087 µIU/mL)	>68 IU/L (>68 mIU/mL)	25	—
35	27 pmol/L (2.08 ng/dL)	0.034 mIU/L (0.034 µIU/mL)	—	12.5	—
42	27 pmol/L (2.08 ng/dL)	<0.02 mIU/L (<0.02 µIU/mL)	—	—	—
49	34 pmol/L (2.62 ng/dL)	<0.02 mIU/L (<0.02 µIU/mL)	>68 IU/L (>68 mIU/mL)	—	2
56	38 pmol/L (2.93 ng/dL)	<0.02 mIU/L (<0.02 µIU/mL)	—	—	2
59	21 pmol/L (1.62 ng/dL)	<0.02 mIU/L (<0.02 µIU/mL)	—	—	2
64	11 pmol/L (0.85 ng/dL)	<0.02 mIU/L (<0.02 µIU/mL)	>68 IU/L (>68 mIU/mL)	37.5	2
71	18 pmol/L (1.39 ng/dL)	0.073 mIU/L (0.073 µIU/mL)	—	37.5	2
78	21 pmol/L (1.62 ng/dL)	<0.02 mIU/L (<0.02 µIU/mL)	49 IU/L (49 mIU/mL)	37.5	2
90	24 pmol/L (1.85 ng/dL)	<0.02 mIU/L (<0.02 µIU/mL)	—	37.5	2
101	19 pmol/L (1.47 ng/dL)	<0.02 mIU/L (<0.02 µIU/mL)	6.1 IU/L (6.1 mIU/mL)	37.5	2
115	18 pmol/L (1.39 ng/dL)	0.04 mIU/L (<0.04 µIU/mL)	2 IU/L (2 mIU/mL)	37.5	2
123	12 pmol/L (0.93 ng/dL)	5.1 mIU/L (5.1 µIU/mL)	—	—	—
136	13 pmol/L (1.00 ng/dL)	2 mIU/L (2 µIU/mL)	—	—	—

Values in parentheses are conventional units. Thiamazole was divided into 2 equal daily doses.

Abbreviations: FT4, free T4; TRAb, TSH receptor antibody.

## Treatment

The patient was started on oral levothyroxine at a dose of 31.25 μg/day (≈10-15 μg/kg/day) on day 3 of life. After initiating treatment, a swift decline in TSH was noted (Table 1).

## Outcome and Follow-up

After approximately 1 month, serum TSH became suppressed and the levothyroxine dose was reduced. Despite dose reduction, serum FT4 concentrations kept rising and TSH remained suppressed. Levothyroxine was therefore completely stopped. TRAb titers remained elevated above the assay's detection limit, and at the age of 7 weeks a diagnosis of TSAb-induced hyperthyroidism was made. The patient was started on oral thiamazole 1 mg twice daily (≈0.5 mg/kg/day). Because of tachycardia, propranolol 2.5 mg twice daily (≈2 mg/kg/day) was added.

Two weeks after starting thiamazole, propranolol could be stopped and levothyroxine 37.5 μg/day was reintroduced due to falling FT4 levels. The patient was successfully treated according to the “block and replace” strategy, with biweekly thyroid function tests and periodic TRAb titer measurements. Preference was given to the block and replace method instead of the titration method to prevent thyroid hormone fluctuations. TRAbs became undetectable at the age of 4.5 months and treatment was stopped. On follow-up until the age of 6 months, the patient remained euthyroid.

## Discussion

We report on a newborn with transient hypothyroidism followed by transient hyperthyroidism caused by maternal TRAbs. The mother was diagnosed with Hashimoto disease and treated with levothyroxine. Despite high TRAb titers, she had never experienced signs or symptoms of hyperthyroidism and did not use ATDs, possibly due to a predominance of TBAbs. An alternative explanation for the absence of (a period of) hyperthyroidism in the mother could be that her thyroid had been destroyed by the Hashimoto thyroiditis and became unable to react to later occurring TSAbs [9]. During pregnancy, no ultrasonographic abnormalities of the fetus were detected. After birth the neonate was found to have high TRAb titers and primary hypothyroidism, which was treated with levothyroxine. After approximately 1 month, biochemical signs of hyperthyroidism ensued and levothyroxine treatment was stopped. Despite stopping levothyroxine, hyperthyroidism persisted and was treated with thiamazole and propranolol. The child was subsequently treated according to the block and replace principle until TRAbs became negative after 4.5 months. After treatment was stopped, the child remained euthyroid.

Offspring of mothers with TRAb-positive autoimmune thyroid disease usually display either transient primary hypothyroidism or transient primary hyperthyroidism, which on rare occasions is followed by (transient) central hypothyroidism [17].

The type of thyroid dysfunction seen in offspring seems to be directly related to the dominant type (ie, blocking or stimulating) of TRAb present in the mother. In pregnant women with a nonfunctional thyroid, for example, due to end-stage Hashimoto disease or after definitive treatment (radioactive iodine or surgery) for Graves’ disease, it can be hard to clinically determine the dominant type of TRAb. Furthermore, most TRAb assays (immunoassays) do not discriminate between TSAb and TBAb, which needs a bioassay [4]. Unfortunately, our laboratory was not able to assess the functional properties of the TBAbs due to the lack of a bioassay.

In neonates born to mothers with elevated TRAb titers, euthyroidism at birth does not exclude the development of thyroid dysfunction at a later stage. In fact, the phenomenon of delayed thyrotoxicosis has been noted to occur as late as 45 days after birth [18]. It is assumed that delayed thyrotoxicosis occurs when the effects of maternal TBAbs and TSAbs are balanced at first but shifts toward TSAbs over time due to a shorter half-life of TBAbs [2, 9, 10, 18, 19].

As noted in the introduction, TRAbs are the main antibody in Graves’ disease but are also found in approximately 10% to 20% of patients with Hashimoto disease [1, 2, 6, 7]. TBAbs seem to be the predominant TRAb in Hashimoto disease and are sporadically associated with transient neonatal primary hypothyroidism [1, 7, 12-15]. Nevertheless, the (delayed) occurrence of neonatal thyrotoxicosis in offspring of mothers with Hashimoto disease due to TSAbs has also been anecdotally reported [11, 18, 20-27]. In contrast to our patient, the thyrotoxic phase in these cases was never preceded by overt hypothyroidism.

Most cases of transient neonatal primary hypothyroidism (followed by transient hyperthyroidism) are caused by maternal ATD treatment for Graves’ disease, which masks the stimulating effects of circulating TSAbs [2]. As the half-life of ATDs like propylthiouracil is short, these children usually do not require levothyroxine supplementation before hyperthyroidism or euthyroidism ensues.

To our knowledge, this is the first report on the sequential occurrence of transient neonatal primary hypothyroidism followed by transient hyperthyroidism caused by maternal TRAbs with mixed properties. We hypothesize that in our case TBAbs were the predominant antibody causing primary hypothyroidism. Over time, the TBAb titer decreased more rapidly than the TSAb titer, ultimately shifting the balance toward TSAbs resulting in thyrotoxicosis.

As the occurrence of neonatal thyrotoxicosis in offspring of mothers with Hashimoto disease due to TSAbs is extremely rare, we do not advice routine measurement of TRAbs in (pregnant) women with Hashimoto disease who have not experienced thyrotoxicosis. TRAb testing in pregnancy is indicated when hyperthyroidism, a history of Graves’ disease (irrespective of treatment), a previous history of delivering an infant with hyperthyroidism, or a known history of thyroidectomy for the treatment of hyperthyroidism in pregnancy is present [16]. If TRAbs are detected in early pregnancy, repeat testing at weeks 18 to 22 is recommended and, if elevated, again at weeks 30 to 34 [16]. If during gestation TRAb titers are ≥3.7 times the upper limit of the reference interval, then close monitoring of the neonate after birth is warranted [1]. The presence of primary hypothyroidism or euthyroidism does not exclude subsequent thyrotoxicosis in neonates with high TRAb titers [1]. Therefore, we advise that neonates with high TRAb titers (≥4 times the upper limit of normal) be periodically monitored (every 10-14 days) until TRAbs become negative, regardless of thyroid function [1].

## Learning Points

- TRAbs are the primary antibodies in Graves’ disease but are also present in a small percentage of patients with Hashimoto disease.
- TRAbs can have blocking (TBAb) or stimulating (TSAb) properties. Both types of antibodies can be present in the same person, and their relative proportions can change over time.
- During pregnancy, maternally derived TRAbs can cross the placenta and cause thyroid dysfunction in the offspring. The type of thyroid dysfunction (ie, hypo- or hyperthyroidism) depends on the properties of the predominant antibody.
- Due to the shorter half-life of TBAbs compared to TSAbs, transient primary hypothyroidism followed by transient hyperthyroidism can occur in offspring of mothers with TRAb-positive autoimmune thyroid disease even in the absence of thionamide use.
- The presence of high TRAb titers in the neonate should prompt physicians to perform regular thyroid function tests, even if initial results are normal, until maternal antibodies are cleared.

## Contributors

M.G. performed data acquisition, designed the tables, and drafted the article; G.A.K. performed data acquisition and reviewed and edited the manuscript; A.S.P.T. reviewed and edited the manuscript.

## Data Availability

Data sharing is not applicable to this article as no datasets were generated or analyzed during the current study.
